# Pre-treatment Strategies for Value Addition in Poultry Litter

**DOI:** 10.3389/fbioe.2020.00477

**Published:** 2020-05-25

**Authors:** Taís Carla Gaspareto, Thamarys Scapini, Bruno Venturin, Deisi Cristina Tápparo, Aline Frumi Camargo, Marco Di Luccio, Alexsandra Valerio, Rafael Favretto, Fabiane Goldschmidt Antes, Ricardo Luís Radis Steinmetz, Helen Treichel, Airton Kunz

**Affiliations:** ^1^Laboratory of Microbiology and Bioprocess, Federal University of Fronteira Sul, Erechim, Brazil; ^2^Western Paraná State University, Cascavel, Brazil; ^3^Department of Chemical and Food Engineering, Federal University of Santa Catarina, Florianópolis, Brazil; ^4^Center for Agricultural Sciences, Santa Catarina State University, Lages, Brazil; ^5^Embrapa Suínos e Aves, Concórdia, Brazil

**Keywords:** nitrogen recovery, biogas, anaerobic digestion, ultrasound, energy

## Abstract

We studied different pre-treatments of poultry litter aiming to add economic value to this residue. Strategies were applied to extract ammonium nitrogen with the aim of allowing its further use as fertilizer, and to promote the hydrolysis and solubilization of lignocellulosic components with the aim of facilitating its subsequent conversion to biogas. Ammonia extraction was performed by solubilization in water in a one-step process and by successive extraction steps (3 times 60 min). Successive extractions presented greater removal of total ammonia nitrogen than did one-step extraction, solubilizing about 36% of the ammonia in water. In parallel pre-treatment using ultrasound was performed to increase carbon bioavailability for anaerobic digestion. Using this tool, 24.7 g kg^−1^ of total organic carbon and 13.0 g kg^−1^ of total reducing sugars were solubilized, employing 10% dry mass sample amount, 100% amplitude ultrasound at frequency of 20 kHz amplitude and 2.5 min of treatment (energy input of 299 ± 7 kJ L^−1^; 3,822 ± 95 kJ kg^−1^). Anaerobic digestion of ultrassound pre-treated biomass was evaluated using a biological biogas production assay, and an increase of 10% of biogas production was obtained compared to untreated samples (147.9 and 163.0 mL g^−1^ for crude and pre-treated PL, respectively). The findings suggest that these are environmentally friendly and sustainable strategies to add economic value to poultry litter, reducing the environmental impacts of improper disposal.

**Graphical Abstract:**
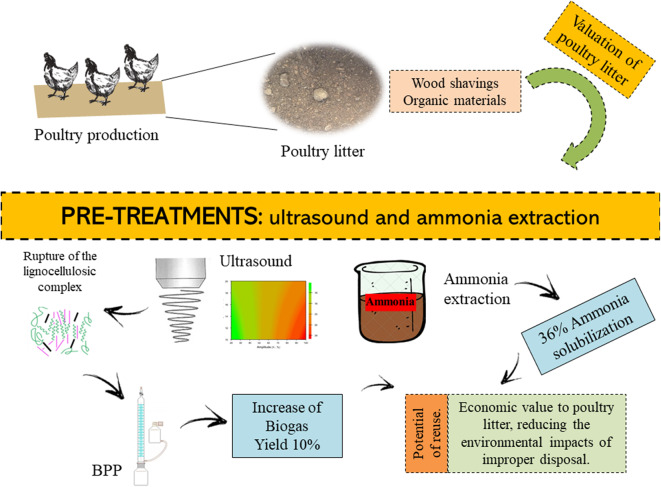


## Highlights

- Strategies for economic value addition on poultry litter were investigated;
- Successive 3 extractions using water solubilised about 36% of ammoniacal nitrogen;
- Nitrogen recovery from poultry litter é feasible using water;
- Ultrasound could be employed as pre-treatment strategy to increase biogas production potential.

## Introduction

The poultry industry has been growing rapidly, spurred by increased consumption of poultry meat and by-products [ABPA (Brazilian Association of Animal Protein), 2018]. Brazil is the second-largest world producer, producing 13.05 million tons of poultry meat annually [(ABPA (Brazilian Association of Animal Protein), 2018)]. In addition to being a lucrative activity, the poultry industry generates large amounts of pollutants (Matusiak et al., 2016), including poultry litter (PL), the primary residue generated in the industry (Burra et al., 2016).

PL is composed of a mixture of organic matter (i.e., manure, wasted feed, feathers, and skin) and the material that composes the litter (i.e., wood shavings, rice husk, or straw). This material has high biotechnological and economic potential, as in anaerobic digestion (AD) for biogas production (Marchioro et al., 2018; Zahan and Othman, 2019). Energetic conversion of poultry residue is considered a good practice because it reduces pollutant generation and could enhance economical profit of poultry farmers (Marchioro et al., 2018).

Nevertheless, the use of PL in biological processes such as AD is a major challenge due to the high concentration of total ammonia nitrogen (TAN) and to the complex structure of PL substrate because of the substantial fraction of lignocellulosic material. Intrinsic difficulties of using this substrate in AD has been recently reviewed by Fuchs et al. (2018). High concentrations of TAN could enhance the concentration of free ammonia (FA) in AD which is toxic because it damages the structure of microorganisms and causes inhibition of its metabolism, compromising their growth (Rajagopal et al., 2013; Tian H. et al., 2018). Several processes have been proposed to minimize FA inhibition, including reduction of ammonia formation precursors or co-digestion with less N-rich materials (Zahan and Othman, 2019), but these approaches could increase the complexity of AD process. Furthermore, the lignocellulosic material in PL, from material that composes the litter, make it recalcitrant to degradation, limiting hydrolysis, and thereby restricting its use in biological processes (Zahan and Othman, 2019). Therefore, to overcome these challenges and increase biogas production from PL, pre-treatment strategies have been used.

Pre-treatment of PL for TAN removal by stripping is reported in some works (Abouelenien et al., 2009; Balaji et al., 2018; Selvaraj et al., 2018). Balaji et al. tested air stripping of ammonia and observed a significant enhance in biochemical biogas potential (BBP) (Balaji et al., 2018). Other simple approach of pretreatment prior to digester unit that could be tested for PL is removal of TAN by water extraction. This procedure has been applied to chicken manure and increased de C/N ratio up to 2.7-folds due to ammonia removal (Böjti et al., 2017). The use of stripping or water extraction enables further nitrogen recovery through reaction of ammonia with sulfuric acid solution to produce ammonium sulfate that can be used as nitrogen fertilizer (Sakar et al., 2019; Busato et al., 2020). Therefore, these economically viable and environmentally sustainable removal and recovery of nitrogen compound techniques are subjects of considerable investigation.

Processes to increase the biodegradability of lignocellulosic structure of PL, reducing its crystallinity and the amorphous components have also been studied for pretreatment and application in AD (Salehian et al., 2013). Usually, the approaches are based on chemical, thermal or enzymatic methods (Costa et al., 2012; Cavalaglio et al., 2018). Costa et al. (2012) evaluated thermochemical pre-treatment in batch experiments as a strategy to improve BBP in PL. In this work, the solubilization of organic carbon was significantly increased with thermochemical pre-treament but authors described that BBP did not increase accordingly which was attributed to possible production of metabolites that could have caused inhibition on methanogenesis. Additionally, chemical and thermal processes are often less attractive due to the costs involved with chemical products and energy consumption.

Ultrasound (US) has been used to increase solubilization of organic carbon on several substrates for AD (Fernandes et al., 2009; Zou et al., 2016; Langone et al., 2018). Is consists of a mechanical process where acoustic waves in frequency of 20 kHz or higher causes the effect known as cavitation in liquid medium that result in bubble formation and subsequent collapse generating high pressure gradients, thereby increasing reactivity and production of free radicals (•OH) due to homolytical clevage of water molecules (Bremner et al., 2011; Bondhoo and Mohee, 2018). Lignocellulosic biomass transformation was reviewed by Den et al. (2018) and US was classified as a greener process for biomass coversion. According to the authors, ultrasound technologies should replace energy-intensive heating to facilitate efficient chemical reactions. In this sense, US is considered a promising pre-treatment technology that could be used without chemical additives or in reduced amounts (Houtmeyers et al., 2014).

Acoustic cavitation facilitates solubilization of organic matter, resulting in larger amounts of substrate readily available for AD, that could accelerate the hydrolysis step and improve final biogas production (Fernandes et al., 2009; Zou et al., 2016). In a comparative study of physical pre-treatment by sludge particle disintegration processes, US demonstrated higher efficiency, increasing the concentration of organic materials and partial deviation of the hydrolytic step in AD (Houtmeyers et al., 2014). This energy source has already been used to produce furanic platforms from cellulose (Santos et al., 2018). Besides published works using US for lignocellulosic material pre-treatment, no publications were found about its use for PL. therefore, this subject is word of investigation as a promising alternative to establish adequate processes for PL valuation.

The objectives of the present study were (i) to evaluate the extraction of ammonia using water in continuous and successive washing, aiming to reduce the inhibition of subsequent biological processes and the possible recovery of nitrogen from the solution; and (ii) to investigate US pre-treatment of the lignocellulosic complex of PL, aiming to improve the application potential in AD. In the extraction of TAN, the relationship between soluble ammonia concentration and pH was evaluated. In the US pre-treatment, the concentration of total reducing sugars (TRS) and total organic carbon (TOC) were evaluated. Finally, the BBP was studied, as well as the kinetic parameters defined by the Gompertz model.

## Materials and Methods

### PL Sampling

PL after 12 production cycles (number of poultry feedlots that where housed using the same litter) and wood shavings were collected at a farm localized in Jaborá, Santa Catarina, Brazil. Wood shaving is the started material used to prepare the poultry house, while PL was collected after its removal from the poultry house. The samples were homogenized and divided in subsamples using a quarter (model H-3980, Humboldt). After were stored in a vaccum bag at −20°C.

### Chemical Characterization of Samples and Analytical Approaches

PL samples were analyzed for determination of total solids (TS), fixed solids (FS), volatile solids (VS), total nitrogen (TN), TAN, phosphorous, potassium, and total carbon (TC), following the Standard Methods for Analysis of Water and Wastewater (APHA AWWA WEF, 2012). TC and TN were determined using an elemental analyzer CHNS-O Flash 2000 (Thermo Scientific) following the manufacturer recommendations.

TAN determination in liquid samples was performed using the colorimetric method in a flow injection analysis system (model 2500, Fialab Instruments, Seattle, USA) using methods adapted from APHA AWWA WEF (2012). TOC was determined in liquid fractions using a TOC analyzer (TOC-LCPH/CPN, Shimadzu, Kyoto, Japan) following the manufacturer recommendations. TRS was determined using the dinitrosalicylic acid (DNS) method and evaluated on a UV-VIS spectrophotometer (model UV-M51, Bel Engineering, Italy) at 540 nm, according to Miller (1959). All analytical parameters were quantified according to standard Methods (APHA AWWA WEF, 2012) and analyses were performed in triplicate following standardized protocols based on good laboratory practices and quality assurance policy.

The results of chemical characterization is expressed as relation of analyte mass in base of natural sample mass. For example, TAN results is expressed as g kg^−1^ and represent mass of nitrogen (as N-NH3) based natural PL mass.

### Evaluation of the Ammonia Solubilization in Water

TAN solubilization experiments were conducted in two experimental ways. In the first way, one step solubilization process was used: 50 g of samples were added to 500 mL of distilled water and maintained under agitation at 130 rpm in JarTest system (model JT102, Milan). The experiment lasted 180 min and samples were collected, placed on filter paper and analyzed for TAN. The pH of suspension was continuously measured using a portable probe (Hanna, model HI 8424).

The second way was a successive solubilization process, where 50 g of samples where placed in a glass becker and 167 mL of distilled water were added then kept under agitation at 130 rpm. The agitation was stopped after 60 min for decantation and separation of liquid and solid fractions, and the solid was submerged again in 167 mL of distilled water and maintained under agitation for 60 min. The procedure was performed three consecutive times and the liquid fraction was sampled for TAN and TOC determination in all collected supernatant samples.

### Evaluation of Increase in PL Lignocellulosic Fraction Bioavailability

#### PL Pre-treatment Using US

PL pre-treatment using US was performed using an ultrasonic processor from Qsonica (Newtown, USA) operating with an output frequency of 20 kHz and equipped with a titanium probe with a tip of 12-mm diameter. The maximum output power of the equipment was 700 W. The absolute energy delivered to the samples is shown on the equipment display, in Joules (J). The energy density (kJ L^−1^) was determined as the absolute energy divided by the volume of the sample treated (40 mL) (Mason et al., 2011).

The experimental design methodology was used to evaluate the effect of US pre-treatment on PL, aiming to evaluate the possibility of increasing the availability of lignocellulosic fraction of biomass. Central composite design (CCD) 2^2^ was developed according to the methodology proposed by (Rodrigues and Iemma, 2014), using a 2^k^ complete factorial scheme, where “k” is the number of independent variables under study, and the assays (factorial points “K”) were determinate by the exponential expression (2^k^ = “K”).

In this study, the experimental design was used to evaluate the dry mass (10 to 20%) and US amplitude (20 to 100%), resulting in a CCD 2^2^ with four assays (levels −1; +1), and three central points (level 0) to evidence the reproducibility of the process and possible to determine the standard error. The dependent variables evaluated were TOC and TRS. The samples were exposed to US for 2.5 min. For the experiments, 4, 6, and 8 g of PL (corresponding to 10, 15, and 20% of solids, w) were transferred to a 100 mL glass becker and 40 mL of deionised water was added.

After US pre-treatment, the samples were centrifuged (model Universal 320, Hettich) at 3,500 rpm, 5 min, and the supernatant was used for the determination of TRS and TOC. A solid fraction obtained after US pre-treatment was dried in an oven at 105°C and analyzed using scanning electron microscopy (SEM) on Hitachi equipment (model TM3030). SEM analyze was performed in a scanning electron microscope (SEM/EDX, TM-3030, Hitachi) in samples: crude PL (control) and the CCD 2^2^. Samples were fixed with carbon tape on aluminum holders and then coated by 18 gold sputtering (Ernest Fullam) for 30 s. All SEM analysis were carried out at 15–19 kV.

#### Increased Energy Applied to PL

Assays of increasing the exposure time of the samples to US were performed, aiming to increase the energy applied in the PL and enabling greater degradation of the lignocellulosic structure. The conditions pre-established by CCD 2^2^ were used for assays, with 3.4 g (dry mass) of samples in 27 mL of distilled water and US at 100% amplitude. The exposure times varied from 5 to 20 min. The assays were conducted in triplicate and the responses were expressed as TRS and TOC.

### Biochemical Biogas and Methane Potential Assay

The biochemical biogas potential (BBP), in batch tests, were used to evaluate the PL biogas recovery and pretreatments efficiency on the kinetic parameters. The bioassays were performed according (VDI 4630, 2016) using 250 mL glass digester coupled to gruaduated 500 mL eudiometer tube.

Reactors were filled with 200 g of previously acclimated mesophilic inoculum and mixed with 4 g of substrate of crude PL or 4, 6, or 8 g of PL after US pre-treatment (as described before). The proportion of substrate/inoculum was performed according to the guideline VDI 4630 (VS_substrate_/VS_inoculum_ ≤ 0.5). After the reactor was coupled to the eudiometer tube, sealed and the headspace flushed using N_2_. The reactor was kept at 37°C in a thermostatic bath. The biogas production was measured daily by sealing liquid level displacement (sealing solution according, DIN Deutsches Institut für Normung., 1985) until stability (dV/dt < 1% of cumulative volume), usually for 30 days. Assays were performed in triplicate. At the same time control tests are carried out to evaluate the biogas production from the inoculum, as negative control (blank), and a second with high purity microcristalline cellulose (Type 20, Sigma-Aldrich) as positive control substrate. The data analysis and quality control were performed as recommended as suggested by Holliger et al. (2016). The CH_4_ content in biogas was determined by infrared absorption sensors using BIOGAS 5000 (Landtec, USA).

The enrichment and acclimated mesophilic inoculum was obtained from a lab scale bioreactor described by Steinmetz et al. (2016). It consisted of equal parts of dairy manure mixed with UASB sludge from an industrial food wastewater treatment plant and mixed with UASB sludge from swine manure wastewater treatment plant. The inoculum reactor was maintained under mesophilic conditions (37 ± 1°C) and previously feed with PL for acclimation. Seven days prior to the start of the experiments, feeding of inoculum was interrupted to reduce the baseline effect (Steinmetz et al., 2016).

### Gompertz Model

The Gompertz model (Equation 1) was applied to determine the kinetic parameters of substrate degradation for biogas production (Ware and Power, 2017).

(1) $$\begin{array}{r} M\left(t\right)\;=\;A*\text{exp}\;\left(-\text{exp}\;\left(\left(\frac{{\text{r}}_{\text{m}}}{\text{A}}\right)*\left(\lambda -\text{t}\right)*\text{e}1+1\right)\right) \end{array}$$

where “M(t)” is cumulative biogas production (mL g^−1^), in time t (d), “A” is the ultimate biogas production (mL g^−1^ . d^−1^), “r_m_” is the maximal biogas rate (mL g^−1^ d^−1^), and “λ” is lag phase in days (d). The comparison of parameters of the non-linear regression model was developed using software Statistic 8 (trial version).

### Statistical Analysis

Statistical analysis of the various responses was performed using the online Protimiza Experimental Design (experimental-design.protimiza.com.br). Results were expressed as mean ± standard deviation. Analysis of variance (ANOVA), effects and means comparison test (Tukey) were performed, and the confidence level used in the experiments was 95% (*p* < 0.05).

The kinetic parameters data is expressed as normalized volume (273 K and 1,013 hPa) and based in the VS mass content from the natural substrate.

## Results and Discussion

### Characterization of PL

Table 1 presents the characterization of PL. The results indicate a C:N ratio of 7.74, considered low for fermentative processes such as AD, and may limit the application of this material (Rajagopal et al., 2013). Furthermore, because it is a material with high content of lignocellulosic compounds, the C:N ratio may not be representative of carbon bioavailability but part of carbon concentration is also from poultry manure. A PL after 12 production cycles was used and therefore this material presents considerable accumulation of manure. However, probably most of the carbon is in the recalcitrant structure that composes the wood shavings and is not available as a substrate for microorganisms. The effective C:N ratio may be lower than the nominal rate, requiring processes to reduce recalcitrant materials (Shen and Zhu, 2018; Chaump et al., 2019). The high TN and TAN present in PL can causes inhibition during AD process. In the AD hydrolysis step, organic nitrogen is converted to TAN; in alkaline pH conditions, FA (as NH_3_) is formed. This chemical species can be toxic to microorganisms due to microorganism cell membrane direct permeation (Kunz and Mukhtar, 2016; Tian H. et al., 2018). On the other hand, TN and TAN on PL makes it a compelling source for ammonia recovery for fertilizing or other purposes. Studies have developed proposals to mitigate the harmful effects of high TAN concentrations in PL to enable its application in processes such as AD (Rajagopal et al., 2013; Marchioro et al., 2018). However, in both cases, remains a need for low-cost and simple operation solutions.

**Table 1:** Chemical composition of PL used in pre-treatment experiments (concentrations are mean ± standard deviation, *n* = 3).

**Parameter**	**Concentration**
TS (g kg^−1^)	781.9 ± 15.2
FS (g kg^−1^)	407.1 ± 11.0
VS (%, m m^−1^)	374.8 ± 9.2
TC (g kg^−1^)	170.3 ± 2.1
TN (g kg^−1^)	22.0 ± 0.5
C:N	7.7:1
TAN (g kg^−1^)	5.3 ± 0.2
Phosphorus (g kg^−1^)	12 ± 0.5
Potassium (g kg^−1^)	24.3 ± 0.7

The concentrations of phosphorus and potassium were 12.0 and 24.3 g kg^−1^, respectively, characteristic of this kind material and representative of its potential for fertilizing purposes. The concentration of VS was 37.48% (w w^−1^) that corresponds to 48% of TS (dry mass).

### Extraction of TAN Using Water

To develop a simple method that considers the solubility of ammonia in water, the extraction of TAN present in PL was evaluated in two configurations: extraction in one step and successive steps extraction with exchange of aqueous medium.

**Figure 1:**
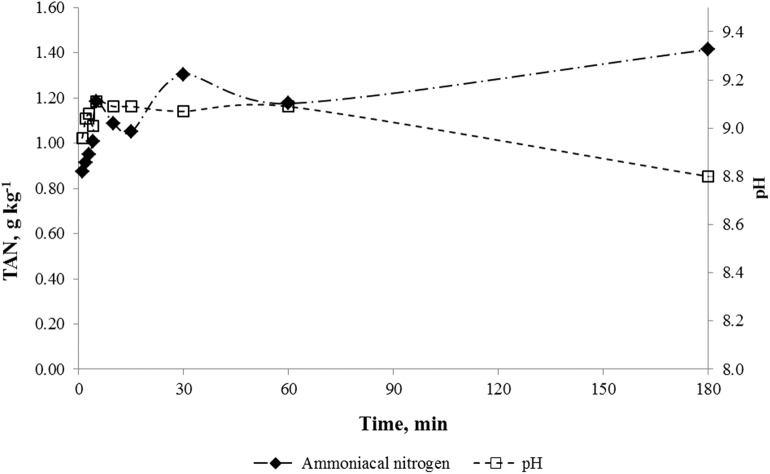
Solubilization of ammoniacal nitrogen from PL in one extraction step experiment. Note: Experimental error for all assays was <5%.

Figure 1 shows the results of TAN in one-step extraction of PL for 180 min compared to suspension pH.

TAN extracted from PL into solution reached 1.31 g kg^−1^ after 30 min of extraction, corresponding to 24.5% of TAN of PL sample (Table 1). This value remained almost constant up to 180 min of extraction (solubilization of 26.6% of ammoniacal nitrogen). The pH in solution remained close to 9.0, highlighting the alkaline characteristics of PL. The solubilization of ammonia in water was expected because of its solubilization characteristics. This chemical species is highly soluble in water, where at 25°C and 1 atm the solubility of NH_3_ é 34%, producing an alkaline solution due to chemical equilibrium displacement to NH_3_ (Félix and Cardoso, 2004; Kunz and Mukhtar, 2016).

The experiment of one-step extraction was maintained up to 70 h (results not shown); we observed that, after 24 h, the concentration of TAN on solution began to decrease, with the concomitant reduction of pH. This behavior is probably related to NH_3_ volatilization.

To increase ammoniacal nitrogen solubilization from PL, successive extraction experiments were carried out. Ammoniacal nitrogen concentrations after each extraction were 1.21, 0.42, and 0.27 g kg^−1^, respectively, corresponding to 36% of TAN in PL (Table 1). Compared to the maximum efficiency of solubilization obtained in one-step experiment (26.6%), it could be concluded that successive extraction was more effective for TAN solubilization. Böjti et al. (2017) used water extraction for poultry manure pre-treatment and obtained in increase C:N ratio of 2.7-folds and observed a reduction of TN of 53.75 to 21.99 mg kg^−1^. In the present work the C:N ratio from 7.7 to 8.5. However, PL is a more complex substrate than poultry manure due to the presence of lignocellulosic material. Additionally, the concentration of TN in the material used in present work is much higher than in above cited work (22.0 ± 0.5 g kg^−1^, Table 1).

The results obtained on solubilization experiments suggest that this approach could be an efficient and low-cost tool for PL pre-treatment. The liquid fraction obtained after solubilization, rich in ammoniacal nitrogen (TAN concentration in solution around 400 mgN L^−1^), could be employed for nitrogen recovery for fertilizer or other uses of ammonia rich solutions (Zhang et al., 2020).

Based on this information, a preliminary assay of biogas recovery after PL water extraction was carried out (Supplementary Data). After washing process the BBP of the solid fraction did not change significantly. However, after the washing process, the lag phase was suppressed (λ from 0.5 to <0.1 d), there was observed an increase in the maximum biogas production rate (r_m_ from 15.8 to 25.6 mL g^−1^ d^−1^) and observed acceleration of the anaerobic digestion process (r_m_ day occurrence reduce from 3.2 to 1.6 d).

### US Action on Pre-treatment of PL Lignocellulosic Fraction

The lignocellulosic structure of PL is difficult to degrade using biological processes, limiting its application to biotechnological processes due to its recalcitrant characteristics (Marchioro et al., 2018). Nevertheless, using pre-treatment of the lignocellulosic structure, it is possible to increase the potential of this biomass for application in fermentative processes (Bondhoo and Mohee, 2018; Den et al., 2018). To determine whether the US effect would be enough to improve the availability of the lignocellulosic structure of the PL, an experimental design was constructed evaluating the influence of the dry mass of the residue on the system and the US amplitude.

Table 2 presents the matrix of experimental design CCD 2^2^, where the response variables were TOC solubilization and TRS.

**Table 2:** CCD 2^2^ presented with codified and real values, for poultry litter TOC solubilization and TRS after US pre-treatment.

**Assay**	**US Amplitude (%)**	**TS (%)**	**TOC (g kg^**−1**^)**	**TRS (g kg^**−1**^)**	**Energy (kJ L^**−1**^)**	**Energy (kJ kg^**−1**^)**
1	−1 (20)	−1 (10)	14.1	6.5	85 ± 1	1090 ± 4
2	1 (100)	−1 (10)	24.7	13.0	299 ± 7	3822 ± 95
3	−1 (20)	1 (20)	16.4	6.6	79 ± 1	1012 ± 16
4	1 (100)	1 (20)	21.1	8.3	226 ± 5	2891 ± 66
5	0 (60)	0 (15)	16.9	9.4	180 ± 1	2300 ± 9
6	0 (60)	0 (15)	20.4	8.2	181 ± 3	2312 ± 34
7	0 (60)	0 (15)	19.9	8.2	180 ± 2	2301 ± 30

Using statistical analysis with 95% confidence for the response variables (TOC solubilization and TRS), it was observed that the US amplitude (%) was the only significant independent variable for increased carbon solubilization after pre-treatment. The TRS concentration was influenced by both response variables. Based on these results, mathematical models (Equations 2 and 3) were proposed to determine TOC (Y1) and TRS (Y2) in the system, being US amplitude (x_1_) and dry mass (x_2_), increasing the possibility of valorization of PL through fermentative processes such as AD.

(2) $$\begin{array}{r} Y1\;=\;19,08\;+\;3,82\;x_{1}\;-\;0,31\;x_{2}\;-\;1,44\;x_{1}\;x_{2} \end{array}$$

(3) $$\begin{array}{r} Y2\;=\;8,61\;+\;2,06\;x_{1}\;-\;1,16\;x_{2}\;-\;1,20\;x_{1}\;x_{2} \end{array}$$

**Figure 2:**
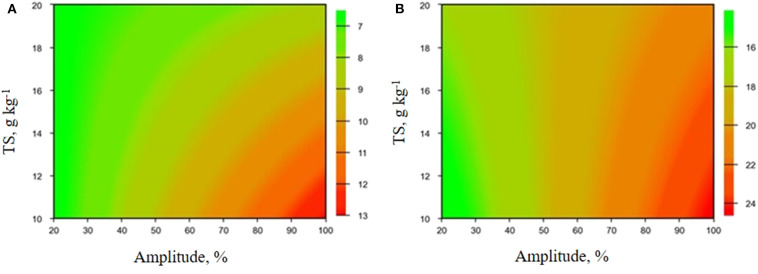
Influence of TS and US amplitude on **(A)** TRS concentration and **(B)** TOC solubilization in PL pre-treatment.

Contour curves (Figure 2) were generated after validation (*p* < 0.05) of a mathematical model for the analysis of variance (ANOVA), with a determination coefficient (*R*^2^) of 90.3% for TOC and 96.6% for TRS. When evaluating the contour curves, it was observed that, regardless of the amount of dry matter, the TOC solubilization increased with increasing US amplitude. A similar phenomenon was observed for TRS, where higher US amplitudes and lower dry mass resulted on the highest yields. The optimal region determination was with maximum amplitude (100%) and the lower amount of dry matter (10%), where TRS and TOC obtained were 13.0 and 24.7 g kg^−1^, respectively. US amplitude is related to energy applied to the reaction system. Therefore, it was observed that the application of more energy was favorable to increase soluble organic carbon levels.

In the biogas production, the presence of higher bioavailable carbon concentration facilitates the transfer of electrons between fermenting bacteria and methanogenic archaea, accelerating the assimilation of acetate by acetoclastic methanogens that are the predominant microorganisms in AD and are responsible for producing 60 to 70% of methane (Biton, 2010; Yun et al., 2018).

Increased TRS concentration may result from the destruction of the lignin-carbohydrate complex, causing the release of sugars from the biomass structure. US pre-treatment may increase the accessibility of microorganisms and enzymes to structures with easy biological degradation, and this result becomes relevant for application in fermentative processes and may be indicative of improvement in hydrolysis steps and reaction rate of these substrates (Shen et al., 2018).

The US pre-treatment on assay 2 resulted in TOC of 24.7 ± 0.7 g kg^−1^ and corresponds to 14.5% of total carbon present in raw PL (170.3 g kg^−1^). TOC in supernatant obtained in water extraction experiments was 16.5 ± 0.5 g kg^−1^ which about 33% lower than TOC solubilised by US. The increase in US energy applied has been related to increase in bioavailable TOC of biomass (Garoma and Pappaterra, 2018). The US energy applied in assay 2 was 299 ± 7 kJ L^−1^, similar to previously reported values (Pérez-Elvira et al., 2014).

The higher energy applied to the system by the increase of US amplitude increased the concentrations of TOC. Assuming that the increases of TOC and TRS are partially related to the degradation of the lignocellulosic complex of the PL (considering that feces and other no lignocellulosic components of PL are easier to solubilise), it can be inferred that the increase of energy applied to the system would cause an even greater degradation of this fraction. Therefore, experiments were carried out to increase the amount of energy applied to the system by applying US for longer times. The results are presented in Table 3.

**Table 3:** TOC and TRS results for increased energy applied to PL by increasing US exposure time.

**Assay**	**Time (min)**	**TOC (g kg^**−1**^)**	**TRS (g kg^**−1**^)**	**Energy (kJ L^**−1**^)**	**Energy (kJ kg^**−1**^)**
U1	5	34.4^a^ ± 0.4	16.5^a^ ± 2.1	819^a^ ± 25	1044^a^ ± 306
U2	10	36.5^a, b^ ± 4.9	15.0^a^ ± 5.7	1671^b^ ± 71	21307^b^ ± 962
U3	15	40.5^b, c^ ± 3.7	13.0^a^ ± 1.4	2299^c^ ± 119	29355^c^ ± 1471
U4	20	45.4^c^ ± 1.2	17.0^a^ ± 1.4	3268^d^ ± 48	41647^d^ ± 683

*Equal lowercase letters in same column do not differ statistically from each other by Tukey test (95% confidence level)*.

The sample pre-treated for 20 min (U4) expressed the highest TOC concentration (45.4 g kg^−1^). The effectiveness of US on organic compounds solubilisation was verified compared to crude sample. The longer US application time (higher energy applied) results in a higher TRS solubilisation.

### Scanning Electron Microscopy

**Figure 3:**
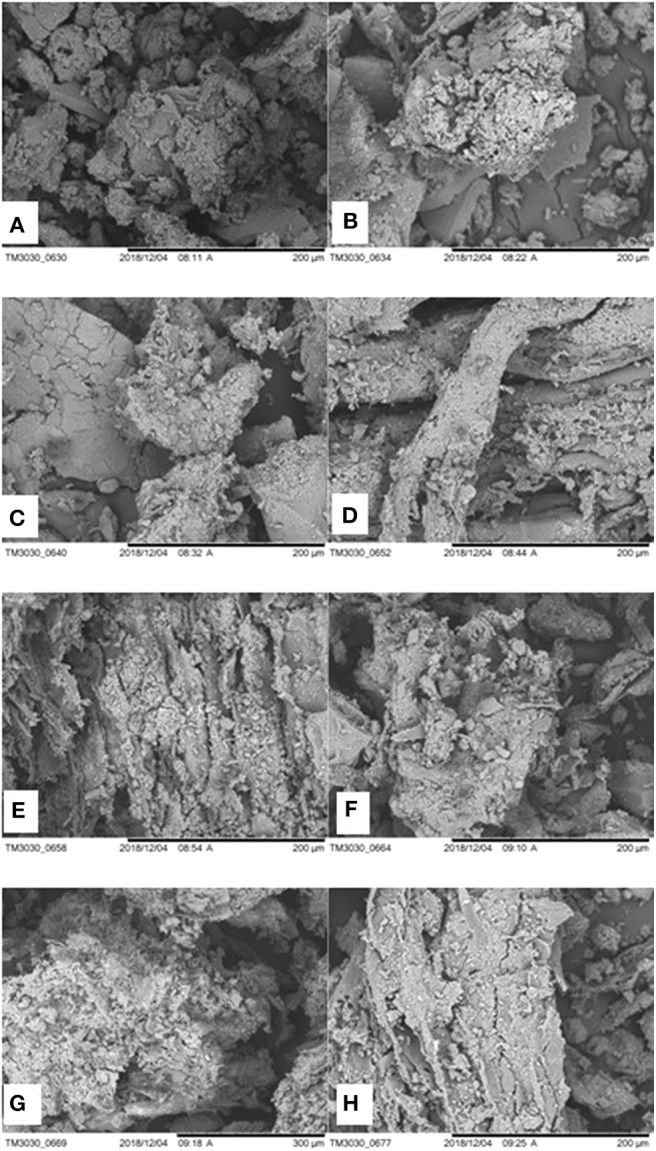
SEM images of crude PL and assays after US pre-treatment. Where: **(A)** crude PL; **(B)** Assay 1; **(C)** Assay 2; **(D)** Assay 3; **(E)** Assay 4; **(F)** Assay 5; **(G)** Assay 6; **(H)** Assay 7.

Alterations in PL structure were evaluated using SEM images of the untreated substrate and assays of US pre-treatment of CCD 2^2^ (Figure 3).

SEM showed that the structure of the PL changed after US pre-treatment, showing regions with greater disintegration and smaller particles, compared to the crude material. It is interesting to note the heterogeneous surface with particles of various sizes on samples after US treatment.

The sample of crude PL had less rough surface than all other conditions studied, maintaining its preserved surface structure. In all pre-treated samples, it was observed greater distribution and increase of surface roughness compared to control. This observation was also reported in the study of Zou et al. (2016). This change in physical structure may be linked to the effect of US cavitation that acts on the sample via shear forces (Lippert et al., 2018). Mason et al. (2011) found that the collapse of cavitation bubbles in water and near solid materials can lead to changes in the surface, resulting in erosion and abrasion.

SEM results corroborated the results of TRS and TOC release assays, and those results presented by Zou et al. (2016). These authors studied the effect of US pre-treatment on corn straw and bovine manure and reported that sonication reduced particle diameter and increased corn straw roughness, in addition to making the surface of bovine manure more distributed.

### BBP of PL After US Pre-treatment

BBP was performed to determine changes in biogas production in the PL sample after US pre-treatment. Samples of CCD 2^2^ and US energy amplification assays were used in this study.

Based on the cumulative biogas production from the BBP assays performed, the kinetic parameters (ultimate biogas production potential, maximum velocity of biogas production, and day of occurrence of the maximum velocity of biogas production and duration of lag phase) were calculated using the modified Gompertz model. The results are presented in Table 4.

**Table 4:** BPP and kinetics parameters using Gompertz model for PL crude and pre-treated with US.

**Assay**	**Amplitude (%)**	**Dry mass (%)**	**A (mL g^**−1**^)**	**r_**m**_ (mL g^**−1**^ d^**−1**^)**	**r_**m**_ day (d)**	****λ** (d)**	***R*^2^ (%)**
Crude litter	–	–	147.9	20.0	3.3	0.3	98.0
1	−1(20)	−1 (10)	155.3	14.9	3.5	0.0	96.4
2	1(100)	−1 (10)	163.0	20.9	2.9	0.0	97.6
3	−1 (20)	1 (20)	146.7	22.2	2.8	0.6	99.3
4	1 (100)	1 (20)	153.0	21.9	2.6	0.0	98.3
5	0 (60)	0 (15)	134.1	24.7	2.4	0.0	99.1
6	0 (60)	0 (15)	143.0	25.4	2.3	0.0	98.8
7	0 (60)	0 (15)	140.3	24.7	2.3	0.0	98.2

*Where: A = maximum BPP, r_m_ = maximum biogas production rate, r_m_ day = day of occurrence of the maximum biogas production rate, λ = lag phase and R^2^ = determination coefficient*.

The maximum BBP for crude PL (without pre-treatment) was 147.9 mL g^−1^ and a biochemical methane production (BMP) of 81.8 mL g^−1^ (CH_4_ 55.3%). The BMP in PL is very variable and depends on the animal nutrition factors, the lying material source (wood chips, rice rusk, straw) and the litter management (reuse cycles and climate variations). Costa et al. (2012) found a methane production of 145 ± 14 L kg^−1^ in samples from Portugal. Markou (2015) found variable BMP (50 to 150 mL g^−1^) in PL samples from Greece. In similar samples from Brazil, Vicente et al. (2018) found BBP of 179, 158, and 117 L per kg for dry mass PL samples with six, seven and eight bed reuse feedlots, respectively. Marchioro et al. (2018) found a BBP of 183 L kg-1 for sample of PL after 12 cycles of broiler production.

When compared to the results obtained after US pre-treatment, assay 2 presented the highest biogas production compared to the others assays (163.0 mL g^−1^), 10.2% higher than crude PL. Assay 2 showed the highest TOC and TRS concentration and, as presented in the previous item, it can be inferred that the US energy enabled the increase of the accessibility of the structure by simplifying and increasing the surface area. These findings suggest that biogas production could be improved by increasing the availability of compounds to anaerobic microorganisms (Cesaro and Belgiorno, 2014).

The maximum biogas production rate (r_m_) was higher in the pre-treated samples than in the crude PL, mainly in assays 5, 6, and 7 (> 24.7 mL d^−1^) using 60% of US amplitude and 15% of dry mass. The exception was assay 1, possibly the result of the low energy applied to the system (126.33 kJ L^−1^), with consequent reduction of facilitated access to the hydrolysis step.

Increased biogas production and production velocity can be inferred from the greater release of easily accessible organic material after pre-treatment. The disintegration of PL particles, observed by SEM, can cause a deviation of the hydrolytic step and consequently greater biogas production. The same was observed in the study of Houtmeyers et al. (2014).

Methane percentage on samples pre-treated by US did not show significant changes in relation to crude PL, on average 63%. These results have been observed in other studies, suggested that US promoted mechanisms that do not necessarily lead to the disintegration and solubilization of organic matter but cause changes in the structure of the organic substrate, possibly causing changes in the reaction rate and velocity of the hydrolysis step (Cesaro and Belgiorno, 2014; Houtmeyers et al., 2014).

US is efficient in reducing biomass particles size, facilitating the access of microorganisms in the degradation of easily assimilable compounds; however, some organic particulates require more aggressive pre-treatment for disruption and are relatively difficult to disrupt mechanically. The combination of chemical substances is also reported to improve lignocellulosic compounds exposure. Nevertheless, chemical additives are not selective and in case of PL, other organic compounds that are important for biogas production would also be degraded (Santos et al., 2018; Tian X. et al., 2018). Therefore, it was inferred from this study that the application of higher energy by increasing the exposure time to the US system could improve biogas production. The results were presented in Table 5, where the conditions employed on assays U1–U4 and results for TOC and TRS have been previously reported (Table 3).

**Table 5:** BPP, methane percentages and kinetics parameters of Gompertz model in samples with higher energy applied in PL pre-treatment by US.

**Assay**	**Biogas**	**Methane (%)**	**A (mL g^**−1**^)**	**r_**m**_ (mL g^**−1**^ d^**−1**^)**	**r_**m**_ day (d)**	****λ** (d)**	***R*^2^ (%)**
U1	154.7 ± 11.0	56.6	153.4^a^ ± 10.4	21.6^a^ ± 1.0	4.1^a^ ± 0.1	1.4^a^ ± 0.1	99.9
U2	140.6 ± 9.0	55.3	139.9^a, b^ ± 8.7	20.0^a^ ± 1.5	3.9^a^ ± 0.1	1.3^a^ ± 0.1	99.9
U3	150.5 ± 7.0	56.7	149.4^a, b^ ± 8.3	21.7^a^ ± 1.1	3.9^a^ ± 0.2	1.3^a^ ± 0.1	99.9
U4	126.0 ± 11.0	58.9	126.3^b^ ± 11.4	19.4^a^ ± 1.6	3.7^a^ ± 0.1	1.3^a^ ± 0.1	99.7

*Where: A = maximum BPP, r_m_ = maximum biogas production rate, r_m_ day = day of occurrence of the maximum biogas production rate, λ = lag phase and R^2^ = determination coefficient. Note: equal lowercase letters in same column do not differ statistically from each other by Tukey test (95% confidence level)*.

Experimental results of BBP were in agreement with theoretical values obtained using Gompertz model (*R*^2^, Table 5). TOC increased with the increase in US time (Table 3) however a result that was different from what was expected the application of larger amounts of energy decreased biogas production as demonstrated by results for biogas obtained on assays U1–U4 (Table 5). This may be related to the occurrence of mineralization phenomena due to the absence of US selectivity (Cesaro and Belgiorno, 2014).

In the PL, in addition to the lignocellulosic material from the wood shavings, there are animal feces that are more easily degradable than lignocellulosic fraction. It is possible that, although the increased US time allowed greater degradation of lignocellulosic material, more easily degraded organic compounds suffered complete degradation (CO_2_ formation) and had volatilized. Cavitation in aqueous solutions causes homolytic cleavage of water molecules with the production of hydroxyl radicals (•OH), one of the most powerful known oxidants. On reaction with HO•, organic compounds rapidly convert into oxidized species that then further degrade into smaller molecules, or that fully degrade to CO_2_ and H_2_O (Bremner et al., 2011).

These findings suggest that, as the concentration of TOC increased with the increase of US time and energy applied (Table 3), it is possible that some of organic compounds were lost and other less biodegradable compounds that remained into solution did not contribute to the same extent for biogas production (Passos et al., 2014). Nominal values obtained for day of occurrence of the maximum velocity of biogas production (r_m_ d) could corroborate with these findings as values decreased with the increase of US energy applied indicating more degradation of lignocellulosic chains. Nevertheless, Tukey test evaluation showed that there was no statistical difference (95% confidence level) among values obtained in different US assays. Values for velocity of biogas production (r_m_, mL g^−1^ d^−1^) and λ (related to duration of lag phase) were also considered statistically equivalent. These data suggest that the application of US during short periods or using controlled amounts of energy could enhance biogas production from PL (Bremner et al., 2011).

### Energy Recovery From PL: A Circular Economy Preview and Possible Pre-treatment Strategies Viability

A simplified analysis to estimate the value addition on pre-treated PL was calculated on basis of processing 1 ton of material. Parameters used in calculating the processing costs are based on the laboratory study with the following conditions: (a) 10 m^3^ of liquid effluent rich in TAN would be generated per 1,000 kg of PL after water washing process; (b) concentration of TAN on liquid extracted fraction is 400 mgN L^−1^, which corresponds to 4 kg on N per 1,000 kg of PL; (c) if applied additional membrane technology process for recovery high purity ammonia, the expected efficiency is 90% (Busato et al., 2020). In this process, ammonia is converted to (NH_4_)_2_SO_4_. Thus, 17 kg of (NH_4_)_2_SO_4_ could be produced/ton of PL; d) finally, considering the price of (NH_4_)_2_SO_4_ as US$ 350/ton, the value of recovered (NH_4_)_2_SO_4_ from one ton of PL is US$5.95.

Furthermore, the biogas from PL can replace the use of firewood for thermal demand during the first days of bird's life, when the animal thermoregulatory system is not yet fully developed. Considering a standard aviary location of 100 m × 12 m size, with the capacity to housing 14,500 broilers, it was estimated that this farm could produce ~100,000 kg of PL after 12 cycles (Marchioro et al., 2018). According the BBP data from crude PL from Table 4, it is possible to produce 14,790 m^3^ of biogas. After US pre-treatment this potential could increase to 17,930 m^3^ of biogas.

Nevertheless, these projections scenarios are based in data from a laboratory evaluation process. It is important to emphasize that complementary full-scale validation are necessary.

## Conclusion

Two different ways of pre-treating PL were evaluated, to value this residue in biotechnological processes of interest. It was found a low C:N ratio and the presence of TAN, both of which may complicate subsequent biological processes. TAN removal from PL by washing with water in a continuous and successive washing system was evaluated. I was found greater removal of TAN in the successive washing process (36 %), enabling the application of PL in TAN recovery processes for industrial use or as fertilizer, and enabling water recovery by reducing the water footprint of the process. It was also evaluated the action of US pre-treatment on the lignocellulosic fraction of PL, obtaining a 10% increase in biogas production. Results of the AD of PL pre-treated with US showed a positive perspective to improve the biogas production, suggesting that the applied technique improves the process and can be tested for other purposes, aiming to add value by improving PL digestibility. The overall goal is to reduce the environmental impacts of direct disposal of the material in the soil using sustainable techniques, without the addition of chemical compounds and with reduced effluent generation.

## Data Availability Statement

The datasets generated for this study are available on request to the corresponding author.

## Author Contributions

TG contributed in the accomplishment of the experiments, treatment and discussion of the data, and writing of the article. TS assisted with the execution of the experiments, treatment and discussion of the data, and writing of the article. BV assisted with the execution of the experiments and treatment and discussion of the data. DT contributed in the accomplishment of the experiments. ML assisted with the execution of the experiments. AV assisted with conducting the experiments. RF assisted with the execution of the experiments. AC assisted with the execution of the experiments. FA contributed in the accomplishment of the experiments using US, treatment and discussion of the data, and writing of the article. RS contributed to the accomplishment of the experiments, treatment and discussion of the data and, writing of the article. HT assisted with the accomplishment of the experiments, treatment and discussion of the data, and writing of the article. AK also assisted in the execution of experiments, treatment and discussion of the data, and writing of the article.

## Conflict of Interest

The authors declare that the research was conducted in the absence of any commercial or financial relationships that could be construed as a potential conflict of interest.

## Abbreviations

AD: anaerobic digestion
BPP: biogas production potential
CCD: Central composite design
FS: fixed solids
PL: poultry litter
TAN: total ammonia nitrogen
TN: total nitrogen
TOC: total organic carbon
TS: total solids
US: ultrasound
VS: volatile solids
SEM: Scanning electron microscopy.
