# Supplementary material for: Pre-treatment Strategies for Value Addition in Poultry Litter
Source: Front Bioeng Biotechnol. 2020 May 25;8:477. doi: 10.3389/fbioe.2020.00477 (PMC7261907; doi:10.3389/fbioe.2020.00477)
Supplement: Supplementary file 1 [file Data_Sheet_1.pdf]

Supplementary data

Manuscript: Pre-treatment strategies for value addition in poultry litter

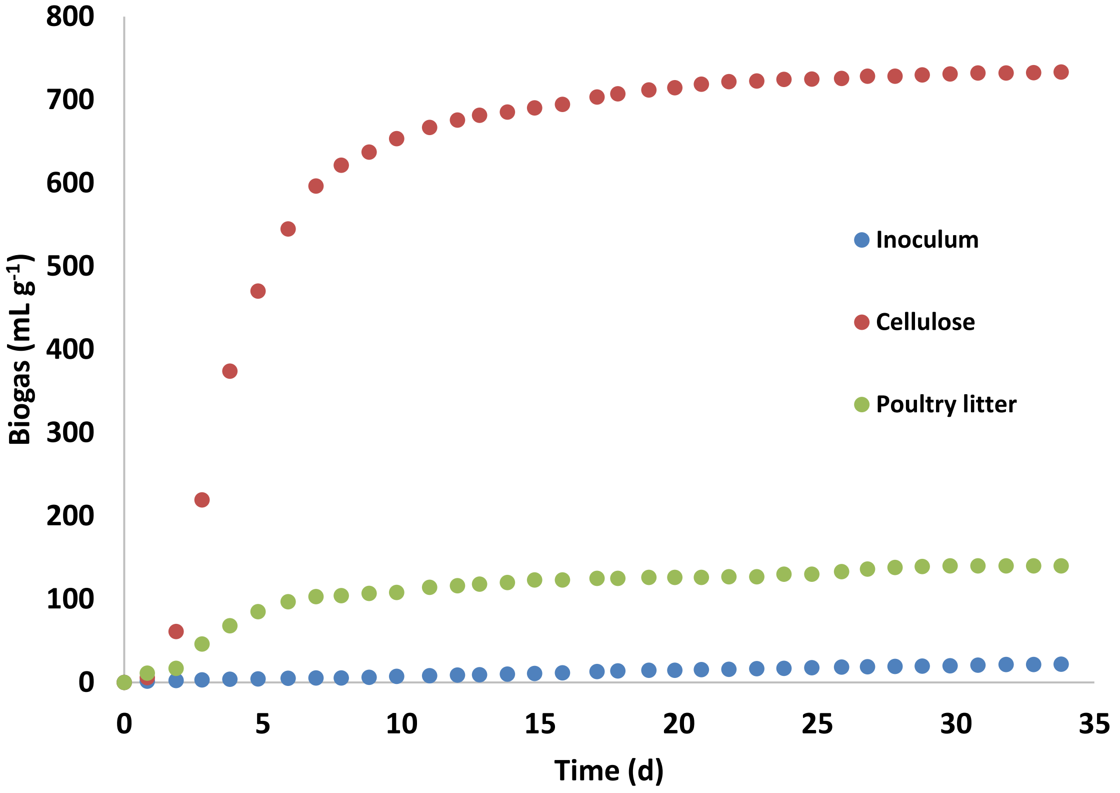


**Figure A.** Profile of cumulative biogas observed for inoculum (negative control or "blank" – blue points), microcristaline cellulose (positive control – red points) and crude poultry litter (sample without pretreatment).

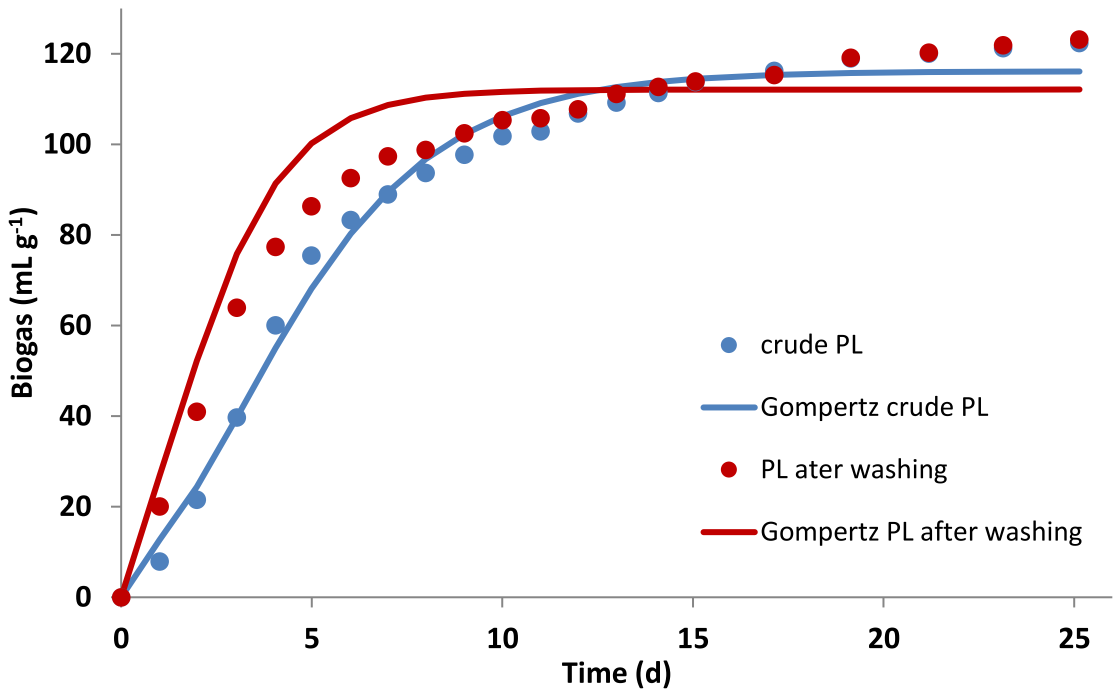


**Figure B.** Profile of cumulative biogas observed for exploratory experimente after PL washing process. Crude PL: blue points; PL after washing: red points; Gompertz model adjusted to crude PL data: blue line; Gompertz model adjusted to data from PL after washing: red line.

**Table A.** The kinetics parameters using Gompertz model for PL crude and after washing exploratory experiment. Where: A = maximum BPP, rm = maximum biogas production rate, rm day = day of occurrence of the maximum biogas production rate, λ = lag phase and R2 = determination coefficient. The means followed by the same letters in the columns do not differ statistically by the Tukey test (5%).

| **Treatment** | **A (mL g$^{-1}$)** | **$r_m$ (mL g$^{-1}$ d$^{-1}$)** | **$r_m$ day (d)** | **λ (d)** | **$R^2$** |
|---|---|---|---|---|---|
| Crude PL | 116.0 ± 24.1$^a$ | 15.8 ± 1.0$^b$ | 3.2 ± 0.6$^d$ | 0.5 ± 0.2$^f$ | 0.9955 ± 0.0083 |
| After washing | 113.1 ± 2.8$^a$ | 25.6 ± 2.2$^c$ | 1.6 ± 0.2$^e$ | 0.0 ± 0.0$^g$ | 0.9391 ± 0.0209 |
